# Potential of Manuka Honey as a Natural Polyelectrolyte to Develop Biomimetic Nanostructured Meshes With Antimicrobial Properties

**DOI:** 10.3389/fbioe.2019.00344

**Published:** 2019-12-04

**Authors:** Elena Mancuso, Chiara Tonda-Turo, Chiara Ceresa, Virginia Pensabene, Simon D. Connell, Letizia Fracchia, Piergiorgio Gentile

**Affiliations:** ^1^Nanotechnology and Integrated Bio-Engineering Centre (NIBEC), Ulster University, Newtownabbey, United Kingdom; ^2^PolitoBIOMed Lab, Department of Mechanical and Aerospace Engineering, Politecnico di Torino, Turin, Italy; ^3^Department of Pharmaceutical Sciences, Università del Piemonte Orientale A. Avogadro, Novara, Italy; ^4^School of Electronic and Electrical Engineering and School of Medicine, University of Leeds, Leeds, United Kingdom; ^5^School of Physics and Astronomy, University of Leeds, Leeds, United Kingdom; ^6^School of Engineering, Newcastle University, Newcastle upon Tyne, United Kingdom

**Keywords:** electrospinning, layer-by-layer assembly, Manuka honey, manofunctionalization, soft tissue regeneration

## Abstract

The use of antibiotics has been the cornerstone to prevent bacterial infections; however, the emergency of antibiotic-resistant bacteria is still an open challenge. This work aimed to develop a delivery system for treating soft tissue infections for: (1) reducing the released antimicrobial amount, preventing drug-related systemic side effects; (2) rediscovering the beneficial effects of naturally derived agents; and (3) preserving the substrate functional properties. For the first time, Manuka honey (MH) was proposed as polyelectrolyte within the layer-by-layer assembly. Biomimetic electrospun poly(ε-caprolactone) meshes were treated via layer-by-layer assembly to obtain a multilayered nanocoating, consisting of MH as polyanion and poly-(allylamine-hydrochloride) as polycation. Physicochemical characterization demonstrated the successful nanocoating formation. Different cell lines (human immortalized and primary skin fibroblasts, and primary endothelial cells) confirmed positively the membranes cytocompatibility, while bacterial tests using Gram-negative and Gram-positive bacteria demonstrated that the antimicrobial MH activity was dependent on the concentration used and strains tested.

## Introduction

Skin and soft tissue infections (SSTIs) are the most common bacterial infections, encompassing a variety of pathological conditions that involve skin and underlying subcutaneous tissue, fascia, and muscles (Esposito et al., 2017). In the USA alone, they account for ~10% of hospital admissions, and they are the most significant cause of morbidity and mortality among hospitalized patients, posing considerable diagnostic and therapeutic challenges (Miller et al., 2015). Moreover, the aging population and the higher number of critically ill patients are playing a crucial role on the incidence of SSTIs, which have increased meaningfully over the past two decades (Tun et al., 2018). The antibiotics has been the cornerstone to prevent bacterial infections. However, several drawbacks, associated with their adoption, have posed serious issues toward their efficacy: (i) bacterial resistance following the release of each new drug, (ii) lack of wide spectrum action, and (iii) reduced function because of the biofilm layers formed by some bacteria (Zhang et al., 2013; Maxson and Mitchell, 2016). For minimizing SSTIs consequences, including the huge financial burden they cause onto the healthcare services and the significant societal costs (Zimlichman et al., 2013), the development of novel and more effective antimicrobial strategies has revealed of paramount importance. Many studies report the importance to inhibit the growth of bacteria in the early biofilm formation stage, thus preventing the infection from starting (Roy et al., 2018). In this regard, the use of technologies at the nanoscale, to develop antibacterial surfaces and coatings, has received great attention within the scientific community, as promising frontier to reduce SSTIs (Pfalzgraff et al., 2018).

Among the nanotechnologies, layer-by-layer (LbL) electrostatic assembly provides to develop multilayered nanocoatings, based on the alternating exposure of a charged substrate to solutions of positively and negatively charged polyelectrolytes (PEs), where a rinsing step is generally included to prevent cross-contamination of the PE solutions. LbL method is an inexpensive and environment friendly, versatile, and simple technique that allows to achieve desired properties and fine control of the coating thicknesses by adjusting the deposition cycle conditions (Gentile et al., 2015a). Furthermore, the driving force of LbL assembly being electrostatic interaction, almost any type of charged specie (e.g., organic molecules or biological macromolecules) can be incorporated into any LbL-treated surfaces (e.g., sheets, fibers, etc.) (Richardson et al., 2015; Ferreira et al., 2019). Within this study, the authors demonstrated that the main advantage of LbL assembly was the relatively small amount of loaded biomolecules/drugs needed to achieve effective concentrations. This has been reported in previous works, where meshes were functionalized with LbL assembly to impart a cascade of nanostimuli and to control the adhesion, proliferation, and differentiation of stem cells with the consequent formation of new bone matrix (Gentile et al., 2017) as well as to effectively deliver the metronidazole drug from oral implant (Gentile et al., 2015b).

Numerous antibiotic-based coatings, constructed via the LbL assembly technique and intended for the development of antibacterial implants, have been investigated so far, given the long-acting stability of LbL film surfaces in comparison to other functionalization techniques (Kruk et al., 2016; Park et al., 2018). However, it has been found that the effect of antibiotics may decrease with time, since antibiotic-resistant bacteria may potentially develop (Li and Webster, 2018). Therefore, the emergency of antibiotic-resistant bacteria remains a big challenge also with the use of LbL assembly method. As an alternative, heavy metals, such as iron, silver, and copper, have been considered as promising PEs for multilayer assembly (Séon et al., 2015; Zhu et al., 2018). However, their high loading has been shown to cause tissue toxicity and impaired wound healing (Guthrie et al., 2012), and bacteria may develop a resistance to metal-based nanoparticles as reported for the silver (Zahin et al., 2019). Moreover, the incorporation of antibacterial peptides and enzymes has also emerged as interesting approach for SSTIs (Pfalzgraff et al., 2018), but their lack of stability during the LbL preparation has limited their application (Zahin et al., 2019).

In the last decade, the antimicrobial activity of different natural compounds has been reported in the literature. As example, chitosan is a well-known biomaterial, obtained from the deacetylation of chitin, produced from the exoskeleton of arthropods, that possesses hemostatic, antioxidant, antitumoral, and bactericidal behavior (Kumar, 2000). Furthermore, oregano, a worldwide used culinary herb, showed antimicrobial and antioxidant properties due to the presence of thymol and flavonoids respectively (Zinoviadou et al., 2009). An alternative natural-based agent, the honey, was proposed in this work in the virtue of its ancient antibacterial properties. Honey has been used to treat infected wounds by indigenous cultures around the globe before bacteria were discovered to be the cause of infections (Molan, 2001). Although some honey varieties have demonstrated to have beneficial effects into infected sites, most modern research has focused on a particular type produced in New Zealand from the nectar of the *Leptospermum scoparium* shrub, called Manuka honey (MH) (Minden-Birkenmaier and Bowlin, 2018). This honey contains the constituents of other honey varieties, but its unique component, methylglyoxal, acts as an additional antibacterial agent (Cokcetin et al., 2016).

The increasing prevalence of data supporting honey's effectiveness as a natural broadband antibacterial agent has encouraged researchers in exploring MH as a wound treatment (Armstrong, 2009) or incorporated in tissue-engineered hydrogels (Bonifacio et al., 2018), demonstrating that MH could significantly reduce the rate of infections on biomaterials and promote fibroblast migration and collagen deposition. In addition, MH could enhance tissue–material integration/regeneration and accelerate healing of the surrounding site (Minden-Birkenmaier and Bowlin, 2018). However, the undesirable cytotoxic effects of high concentrations of honey and its uncontrolled release over time represent two of the greatest hurdles in the development of honey-containing tissue-like substitutes (Sadeghi-Aliabadi et al., 2015). The LbL-assembly approach allows the incorporation of MH with a subsequent and more efficient controlled release of honey from the nanolayers. This strategy offers solutions to the current warning about the potential toxic effects of high honey concentration on myofibroblasts and local mesenchymal-stem cells (Du Toit and Page, 2009). Moreover, the main advantage of this approach is the relatively small amount of honey loaded to achieve a therapeutic outcome, preventing both bacteria resistance and drug-related systemic side effects.

In this work, nanostructured honey-based coatings were deposited on biomimetic electrospun poly(ε-caprolactone) meshes via LbL technique to obtain discrete nanoscale layers to incorporate and to control the MH release with minimal interaction with the biomaterial substrate. After the LbL optimization to achieve appropriate MH release kinetics, the nanocoating was characterized by morphological and physicochemical analyses to evaluate the multilayered deposition while by biological and antibacterial behavior to study the MH efficiency.

## Materials and Methods

### Materials

Poly(ε-caprolactone) (PCL; Mw = 82 kDa), poly(sodium4-styrenesulfonate) (PSS; Mw = 70 kDa), 1,6-hexanediamine (ED), chloroform (≥99.9%), formic acid (≥95%), and sodium acetate buffer solution were purchased from Sigma-Aldrich, UK. Poly(allylamine hydrochloride) (PAH) was supplied from Alfa Aesar, UK, while medical-grade MH (400 mg/kg of methylglyoxal) was purchased from ManukaGuard®, USA. Ultrapure water was obtained by a Milli-Q® Integral system (Merck, Italy). All materials and chemicals were used without further purification.

### Electrospun Membranes Preparation

PCL membranes were fabricated using an electrospinning system (Linari Engineering Srl, Italy). Process and solution parameters were optimized to fabricate defect-free nanofibers with dimensions in the hundreds of nanometers scale. Briefly, PCL pellets were solubilized using a chloroform and formic acid solution (70:30 *v*/*v*) to obtain a 12% *w*/*v* concentration of the PCL solution. For each membrane, 0.6 g of PCL was solubilized in 3.5 ml of chloroform for 1 h under stirring, and then, 1.5 ml of formic acid was added and mixed for 20 min. The spinning process was performed at room temperature, and the spinning parameters were set as voltage of 20 kV, syringe flowrate of 1.5 ml/h, and nozzle–collector distance of 20 cm.

### Electrospun Membranes Functionalization

PCL electrospun membranes (size of 10 × 10 cm and thickness of ≈200 μm) were aminolyzed by dipping into ED solution (0.06 g/ml) for 10 min at 20°C, to graft—NH_2_—to get a positive charge on the surface and then abundantly washed in deionized water and left drying for 24 h. PSS (5 mg/ml), PAH (5 mg/ml), and MH (15, 30, 60, and 120 mg/ml) solutions were prepared by dissolving the PEs in sodium acetate buffer solution (pH 5.3–5.5). The ζ-potentials of these solutions was measured by laser Doppler electrophoresis (Zetasizer Nano, Malvern Instrument, USA). For the LbL assembly, aminolyzed membranes were dipped first into the polyanionic solutions (MH) for 15 min, followed by a washing step in sodium acetate buffer solution for 5 min to remove any unbound PE material. The membranes were then soaked in the polycationic solution (PAH) for 15 min followed by a washing step using the same conditions described before. This dipping process was repeated for eight cycles for creating 16 nanolayers. The samples were left to dry overnight, coded as MH_1.5, MH_3, MH_6, and MH_12, the membranes functionalized using different concentrations of MH (1.5, 3, 6, and 12% *w*/*v*, respectively) in sodium acetate buffer solution (pH 5.3–5.5), while the membrane coated with PSS and PAH as was coded with PSS/PAH (used as control).

### Physicochemical Characterization

Quartz crystal microbalance analyses (QCM-D) were performed with a QSense Explorer device equipped with an open module (Q-Sense, Sweden). Changes in frequency (Δ*f*) and energy dissipation factor (Δ*D*) were monitored at its fundamental resonance frequency (5 MHz) and odd overtones (3, 5, 7, 9, 11, 13). Gold-coated sensors (QSX301, Q-Sense, Sweden) were used and cleaned following manufacture's instruction before use. Immediately after cleaning, a gold-coated sensor was placed in the open module, and 400 μl of ED solution was gently poured on the sensor surface using a micropipette. After 10 min, the ED solution was removed, and 400 μl of sodium acetate buffer solution was poured to remove non-adhered molecules. Then, the LbL process was reproduced by alternating MH and PAH solutions to obtain eight-bilayers, following the same procedure described before.

Morphological analysis of the samples before and after LbL dip assembly was performed by SEM (Philips XL30-ESEM). Specimens were priory sputtered with a thin layer of gold (~10 nm, sputter time of 40 s at 40 mA). All the images were taken at 20 kV and working distance of 10 mm.

Atomic force microscopy (AFM) characterization was performed with a Bruker Icon AFM with TESPA-V2 probes in tapping mode at 320 kHz frequency. The fibers were bonded to a metal stub using two part-epoxy, and the loose fibers above the surface were manually removed to allow probe access to the well-bonded fibers beneath.

X-ray photoelectron spectroscopy (XPS) was performed with Theta Probe (Thermo Scientific, UK) equipped with a microfocused AlKa X-ray source (1,486.6 eV), operated with a 400-μm spot size (100 W power). Process parameters were 200 eV pass energy, 1 eV step size, and 50 ms dwell time in not angle-resolved lens mode. Moreover, high-resolution spectra were acquired with 40 eV pass energy, 0.1 eV step size, and 200 ms as dwell time. Fourier transform infrared spectra were acquired in a wavenumber range of 4,000–550 cm^−1^ using Spectrum Two PE instrument equipped with a horizontal attenuated total reflectance crystal (ZnSe) (PerkinElmer Inc., USA; 4 cm^−1^ resolution and 32 scans).

The amount of MH released from the membranes was analyzed by *in vitro* tests after immersion of 1 × 1 cm membranes in 1 ml of phosphate-buffered saline (PBS, Sigma-Aldrich, UK) solution at 37°C for different time points (up to 4 weeks). The released solution of the soaked membranes was assayed for glucose (Glucose Assay Kit, Sigma-Aldrich, UK) as proposed by Hixon (Hixon et al., 2017).

### Cell Tests

Membranes with a dimeter of 15 cm were treated with Sudan Black (SB, Sigma-Aldrich, UK) to avoid the autofluorescence of the electrospun membranes: 0.3% (*w*/*v*) SB solution was prepared in 70% ethanol, and the membranes were immersed in this solution for 15 min and then rinsed three times in PBS solution and sterilized under ultraviolet light for 30 min (Dai et al., 2016).

Different cell lines were used to test the membranes' cytocompatibility. Human telomerase reverse transcriptase immortalized fibroblasts from non-malignant myoma (T-HESCs, ATCC, CRL-4003™) were cultured from frozen stock in a 1:1 mixture of Dulbecco's modified Eagle's medium and Ham's F-12 medium with 3.1 g/L glucose and 1 mM sodium pyruvate and without phenol red (Sigma-Aldrich, UK) supplemented with 1.5 g/L sodium bicarbonate, 1% ITS+ Premix (Corning, UK), 500 ng/ml puromycin, and 10% charcoal/dextran treated fetal bovine serum (HyClone, US). Healthy skin human fibroblasts, kindly donated by Ana Tiganescu at Saint James's Teaching Hospital in Leeds (isolated from skin biopsies samples obtained from the Tissue Bank repository), were cultured in 1:1 mixture of Dulbecco's modified Eagle's medium with high glucose, Glutamax, and 10% fetal bovine serum. Finally, primary human umbilical vein endothelial cells were isolated from umbilical cord obtained from deidentified term placenta collected from patients who underwent elective Cesarean section between 37 and 39 weeks of gestation. After isolation, 95% purity of endothelial cells was observed, validated morphologically and by immunofluorescent staining for CD31 (DAKO, USA) before seeding on the membranes. Cells were cultured in EBM-2 medium supplemented with EGM-2 Single Quot growth factors (Lonza, USA). All the cell lines were maintained at 37°C in a saturated humidity atmosphere containing 5% CO_2_, and they were subcultured before reaching 60–70% confluence (approximately every 2 days) up to passage 5. All the cells were passaged and seeded on the different meshes at density of 5,000 cells/cm^2^ (~20,000 cells/well in a 12-well plate).

LIVE/DEAD staining (ReadyProbes® Cell Viability Imaging Kit, Molecular probes) was used following manufacturer protocols to determine the viability of cells and to check proliferation of cells exposed for 8 days to medium conditioned with MH (1.3 and 8.3% *v*/*v*) and on the different substrates after 8 days in culture.

### Bacterial Tests

The minimum inhibitory concentration (MIC) of MH to Gram-positive *Staphylococcus aureus* (ATCC 25923) and *Staphylococcus epidermidis* (ATCC 12228) and Gram-negative *Escherichia coli* (ATCC 25922) and *Pseudomonas aeruginosa* (ATCC 10145) was determined according to Wiegand et al. (Wiegand et al., 2008). MH was tested at concentrations ranging from 3.125 to 500 mg/ml. Bacterial suspensions at the concentration of 5 × 10^5^ colony forming units (CFUs)/ml were inoculated into Mueller–Hinton broth in the absence (control) or presence of the different concentrations of honey and the multiwell plates incubated at 37°C for 16–20 h. MIC was identified as the lowest concentration of honey that prevents visible growth of the tested strains as observed with the unaided eye. Assays were conducted in triplicate and repeated twice.

The antimicrobial effectiveness of LbL-functionalized electrospun membranes was assessed using 3-[4,5-dimethylthiazol-2-yl]-2,5-diphenyltetrazolium bromide (MTT)-based colorimetric assay (Tonda-Turo et al., 2018a). Bacterial suspensions at a concentration of 5 × 10^5^ CFU/ml were prepared in Tryptic soy broth. Afterwards, 1 ml of each of these suspensions was used to dip all the membranes, previously sterilized by 30 min ultraviolet treatment, in 5-ml tubes. Samples were incubated at 37°C for 24 h at 120 rpm. At the end of the incubation time, bacteria were harvested by centrifugation at 12,000 rpm for 10 min and incubated for 30 min in 1 ml of MTT working solution containing 0.03 g MTT (Sigma-Aldrich, Italy) in 9.85 ml PBS supplemented with 50 μl of a 20% glucose solution and 100 μl of a 1 mM menadione solution. Bacteria were then harvested by centrifugation at 12,000 rpm for 10 min and resuspended in 1 ml of DMSO/glycine 0.1 M pH 10.2 (7:1) buffer. Finally, absorbance of each sample was measured by spectrophotometric reading at 570 nm wavelength. The percentage of inhibition of functionalized fibers, compared to PCL fibers (controls), was determined as [1 – (Abstreat/Absctrl)] × 100, where Abstreat indicates optical density of functionalized samples and Absctrl corresponds to the optical density of controls.

### Statistical Analysis

All the experiments were performed at least in triplicate. Results were expressed as a mean ± standard deviation, and statistical significance was calculated using analysis of variance (ANOVA). The comparison between two means was analyzed using Tukey's test with statistical significance level set at ^*^p < 0.05, ^**^p < 0.01, and ^***^p < 0.001.

## Results

Electrophoresis measurements were performed, showing that MH-based solutions ranging from 1.5 to 12% *w*/*v* were negatively charged with ζ-potentials from −12.6 to −22.4 mV, while PAH solution was positively charged with a ζ-potential of +14.5 mV. As a control, negatively charge PSS solution was selected to favor complexation due to its strong interaction with PAH. This had a ζ-potential of −18.8 mV. Furthermore, the evaluation of the electrostatic interaction between MH in different concentrations and PAH was investigated by QCM-D measurements, to confirm the formation of the multilayered structures (Tonda-Turo et al., 2018b).

**Figure 1:**
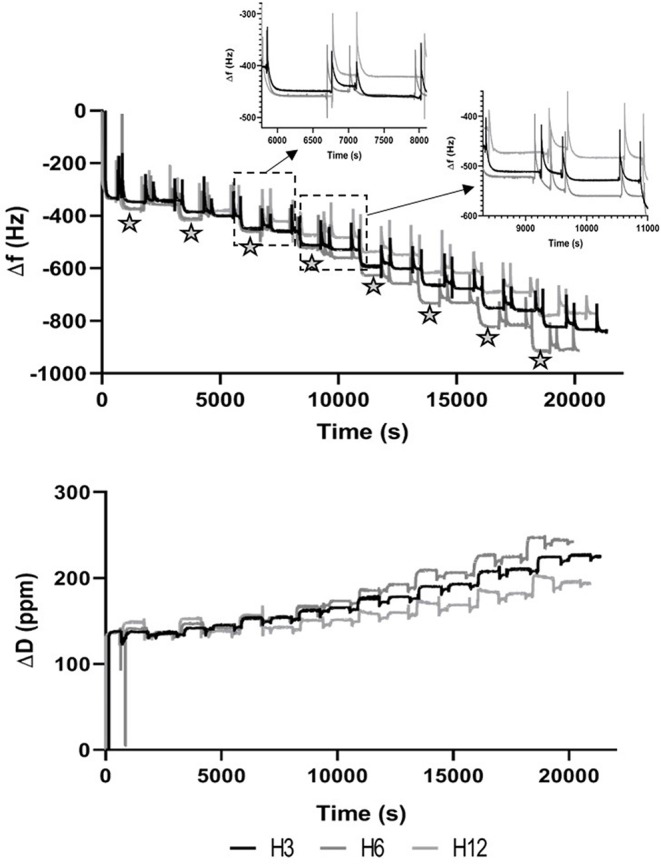
Plot of the fifth overtone of Δ*f* **(A)** and Δ*D* **(B)** vs. time. Stars indicate events associated to the dipping step in Manuka Honey solution.

As shown in Figure 1, the presence of ED solution caused a shift in both frequency and dissipation when NH_2_ groups interacted with the Au surface of the sensor. The subsequent cleaning step partially removed the layer as confirmed by the increase in Δ*f* values (Δ*f* value after cleaning step ≈ −300 Hz) at ~620 s. Then, MH and PAH PE solutions were alternatively flowed across the positively charged functionalized Au crystal for 15 min to simulate the LbL dipping conditions of electrospun membranes. The addition of PEs molecules on the crystal surface was confirmed by a cumulative stepwise response of Δ*f* and Δ*D*, where the black stars indicated the shift caused by the MH-based solutions. The subsequent cleaning step removed the majority of both PEs in the first bilayers. Starting from the third bilayer, the deposition of MH was more evident, particularly for MH_6 and MH_12, while for MH_3, a significant Δ*f* was observed with the formation of the fourth bilayer. Furthermore, the Δ*D* trend confirmed the formation of a thicker layer on the top of the Au crystal as the dissipation factor increased with time.

**Figure 2:**
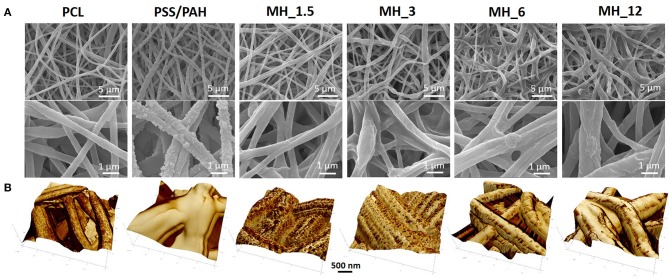
**(A)** Scanning electron microscopy (SEM) and **(B)** atomic force microscopy (AFM) images of the electrospun membranes before and after LbL assembly functionalization.

**Figure 3:**
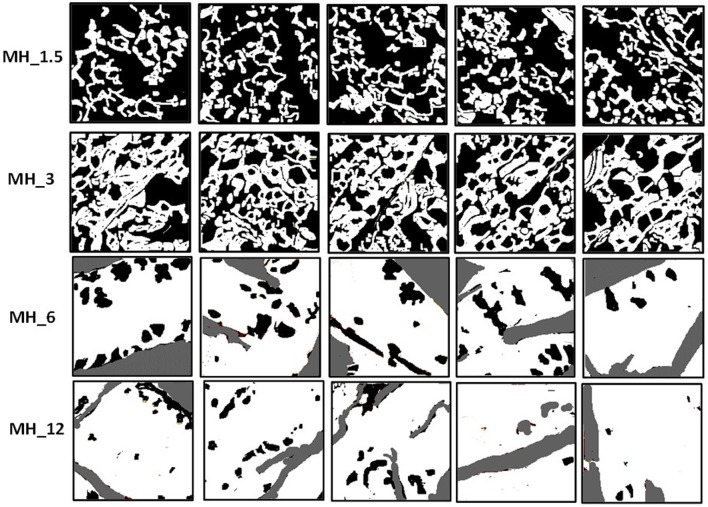
MH adsorption analysis—conversion to thresholded images before image analysis in ImageJ.

The surface morphology of the multilayered coating after LbL assembly was analyzed by scanning electron microscopy (SEM) (Figure 2A). The electrospun membranes presented an average fiber diameter of 0.75 ± 0.22 μm and an intrinsic microporosity. After LbL functionalization, the membranes coated with MH and PAH appeared smooth and uniform, leading to an increase in the fiber diameter, which in turn resulted proportional to the number of bilayers used. The control PSS/PAH-coated membranes showed a formation of irregularly shaped protuberances and a consequently higher surface roughness and fiber diameter in comparison to the bare substrate. Furthermore, AFM analysis (Figure 2B) confirmed the successful functionalization of the membranes, and in addition, it was possible to visualize the extent of the MH coating in detail using the tapping mode phase contrast signal. The clean PCL fibers were smooth to a subnanometer level but, in phase contrast, showed striations of 13.5 nm periodicity around the circumference of the fiber (alternative dark and light stripes of ~7 nm width) (Figure 3).

As with SEM, the PSS/PAH showed a thick, irregular, and continuous coating. MH 1.5–12% *w*/*v* revealed a clear progression of MH coverage. At 1.5% *w*/*v* MH, the coating (bright gold vs. the darker brown PCL in Figures 2B, 3) forms a quite sparse network across the fiber surface. At 3% MH, the strands of this network have thickened and begin to enclose regions of clean PCL. At 6% MH, the network has fused into an almost continuous coating with a small number of patches of PCL fiber visible. These remaining patches were almost entirely gone at 12% MH, and a continuous coating has formed.

**Figure 4:**
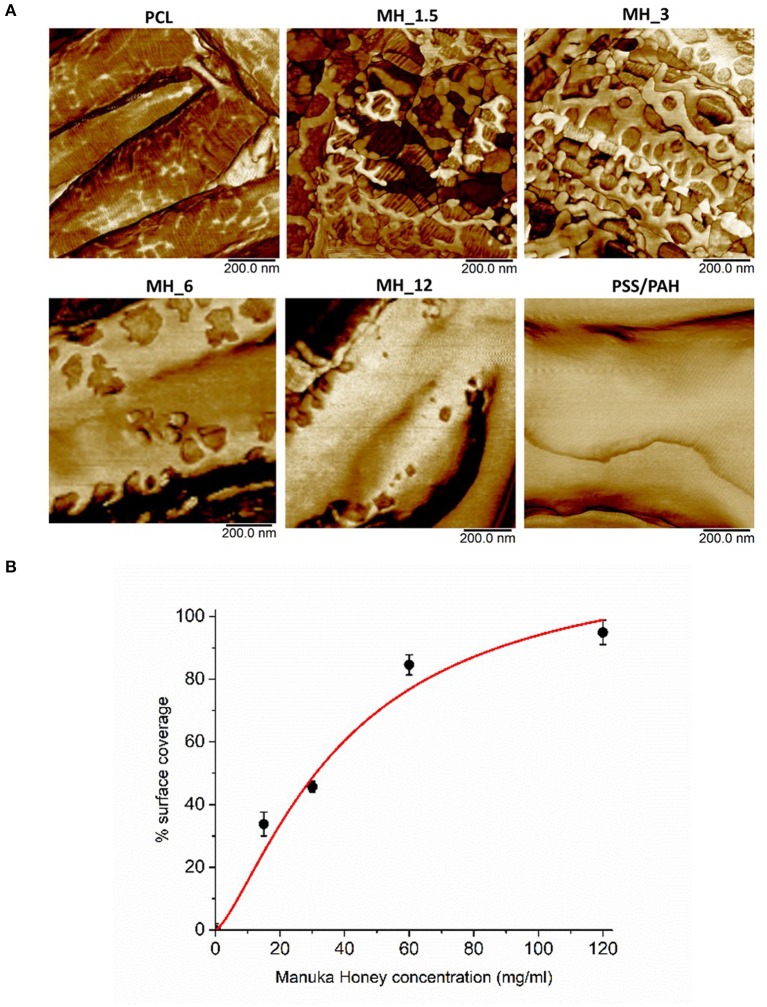
**(A)** High-resolution atomic force microscopy (AFM) phase contrast images. **(B)** Analysis of MH coating coverage from high-resolution AFM images.

The surface coverage of MH visible in the high-resolution AFM phase images (Figure 4A) was analyzed by thresholding to discriminate the coating (Figure 3) and analyzing in ImageJ (NIH) using the histogram function. The resultant concentration vs. coverage response (Figure 4B) is reminiscent of an absorption isotherm, and a Langmuir–Freundlich isotherm (Ratkowsky and Giles, 1990) (allowing for heterogeneous and multilayer films) was used to fit the film growth, giving an absorption rate constant *k* = 6.35 × 10^−3^ L/g.

**Figure 5:**
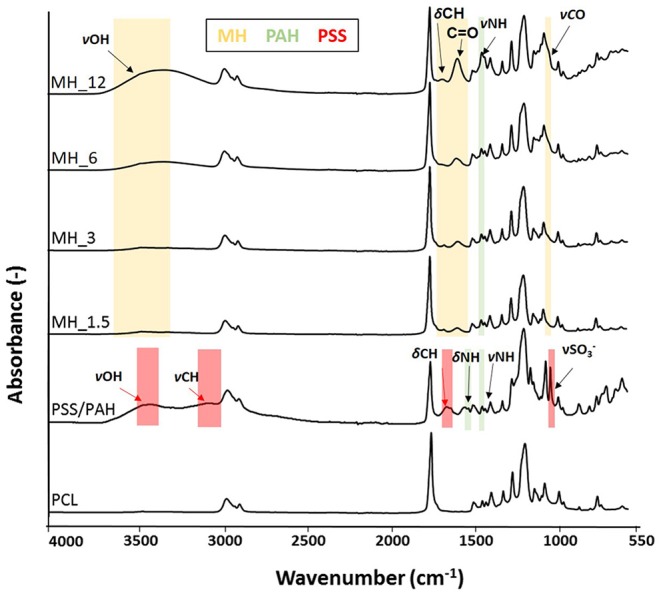
Fourier transform infrared spectroscopy–attenuated total reflectance (FTIR-ATR) spectra of the electrospun membranes before and after layer-by-layer (LbL) assembly functionalization.

Fourier transform infrared spectroscopy–attenuated total reflectance and X-ray photoelectron (XPS) were performed to analyze the surface membranes composition before and after LbL assembly. Particularly, the infrared spectra (Figure 5) revealed the presence of the characteristic chemical bands of the PEs used for coating the PCL electrospun membranes. For the poly(allylamine hydrochloride), the following chemical bands were observed: νN–H stretching (3,360 cm^−1^), alkyl νC–H stretching (2,920 cm^−1^), N–H symmetric and asymmetric scissoring vibrations (1,490 and 1,580 cm^−1^, respectively), and νN–H asymmetric stretching (1,330 cm^−1^) (Gentile et al., 2015b), while the presence of the honey was characterized by different absorption zones dominated by two water bands at 3,300–3,400 cm^−1^ (OH stretch) and 1,641 cm^−1^ (OH deformation). The band from about 1,500–750 cm^−1^ was related to the most sensitive absorption region of the honey's major components, particularly the most suitable region to quantify honey sugar (59–75%) and organic acids. The small peak at 1,110 cm^−1^ corresponded to stretching of the C–O band of the C–O–C linkage, and the peak at ~1,690 cm^−1^ corresponded to C=O stretching (Anjos et al., 2015). However, all the other honey distinctive peaks were overlapped with the other components of PCL and PAH.

**Figure 6:**
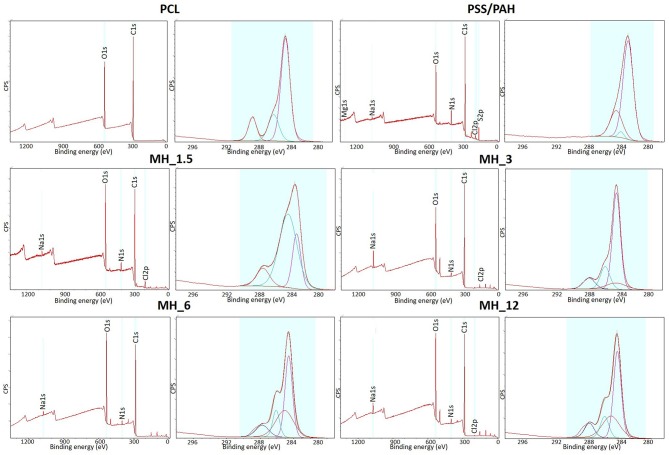
X-ray photoelectron spectroscopy (XPS) survey spectra and high-resolution C1s spectra of the electrospun membranes before and after LbL assembly functionalization.

Figure 6 shows the XPS survey spectra before and after LbL assembly functionalization. The surveys showed N1s peak at 399.5 eV, demonstrating PAH was successfully introduced, while C1s at 285 eV and O1s at 630 eV peaks were characteristics of both PAH and MH. Moreover, the presence of Na1s at 1,070 eV and Cl2p at 200 eV was due to the addition of NaCl in the PE solution for maintaining a stable charge (Rojas et al., 1998). The high-resolution spectra for C1s along with the curve fit showed three peaks attributed to the different carbon oxidation states: (1) 284.7–285.0 eV, (2) 286.8–287.0 eV, and (3) 288.5–289 eV, corresponding to –C–H or –C–C– bonds, to –C–O– bond, and to –C=O groups, respectively. It was observed that these components content varied significantly with the formation of the layers. The concentration of C=O decreased with the formation of the nanocoating because it was characteristic of PCL chemical structure. On the other hand, the component corresponding to C–N bonds increased suggesting the PEs coating. Finally, the high-resolution spectra for N1s revealed the presence of PAH layers in all the coated electrospun membranes (no signal present in bare PCL membrane).

**Figure 7:**
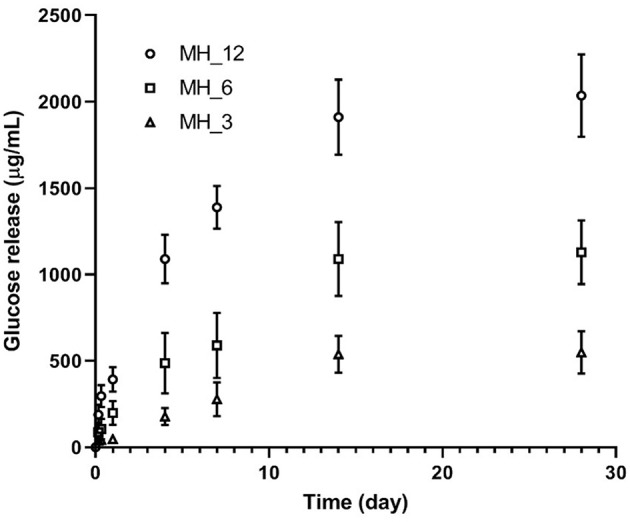
Glucose assay for honey detection.

The release of glucose was measured over 14 days (Figure 7). The LbL-functionalized membranes showed three different stages in the release profile as shown for all the samples, where, as expected, the membrane functionalized with the higher MH content showed the highest glucose released. A burst release was observed with approximately the 10–12% of MH delivered during the initial 24 h of incubation (392.6 ± 47.5 μg/ml for MH_12), followed by a controlled and linear MH release up to 14 days (1.9 ± 0.2 mg/ml for MH_12). At 28 days, no significant change in MH release was noticed for all the samples, probably due to the nanocoating degradation that implied a zero-order release.

**Figure 8:**
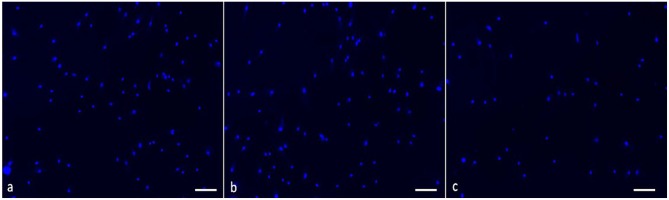
Proliferation of fibroblasts in medium supplemented with Manuka honey at different concentrations. Blue nuclei staining (NucBlue Live reagent) of cells cultured in **(a)** Dulbecco's modified Eagle's medium (DMEM) (control), **(b)** DMEM supplemented with honey 1.3% *v*/*v*, **(c)** DMEM supplemented with honey 8.3 % *v*/*v* was detected using a standard DAPI filter. All the pictures are recorded with magnification 4×, scale bar 100 mm.

When the effect of medium supplemented with MH was evaluated in respect to MH-free medium, the LIVE/DEAD staining showed 100% viable healthy fibroblasts in all the sample: after staining with propidium iodide solution, no signal was detected using a standard tetramethylrhodamine/red fluorescent protein (orange) filter set, meaning that all the cells had an intact plasma membrane (Figure 8). A slower proliferation, however, was observed in high concentrated samples, but not significant in comparison to the control condition.

Then, three different cell types were seeded on all the functionalized electrospun meshes for 8 days. Before seeding the cells, all the substrates were treated with SB. This is a quenching compound commonly used in lipid histochemistry to better visualize biological structure. Using a concentration of 0.3% (*w*/*v*) SB in ethanol that was previously proven to be not toxic and to successfully cancel background fluorescence of polymeric scaffolds (Qi et al., 2017), it was possible to image the live and dead cells after 10 days in culture on most of the samples.

**Figure 9:**
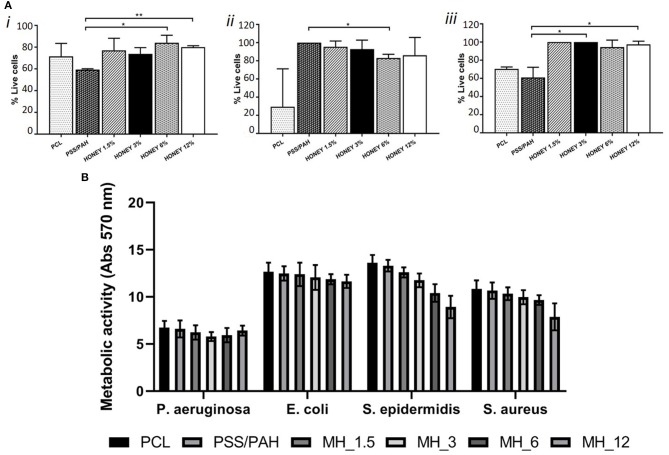
**(A)** Cell viability of dermal fibroblasts (i), immortalized T-HESC (ii) and human umbilical vein endothelial cells (HUVECs) (iii) on PCL, PSS/PAH, Honey1.5, 3, 6, 12%. *Mark significantly different samples. **(B)** Metabolic activity measured by 3-[4,5-dimethylthiazol-2-yl]-2,5-diphenyltetrazolium bromide (MTT) assay of four different strains incubated for 24 h with nanofunctionalized membranes.

Significant differences (*p* < 0.05) were observed only between proliferation on PSS/PAH and MH_6 and MH_12 coated fibers (as shown in Figure 9A). The proliferation of fibroblasts on PSS/PAH sample was lower and statistically different compared to all the other samples. For immortalized T-HESC, the proliferation was significantly decreased on PCL samples, while the concentration of MH did not significantly affect the viability. Significant higher proliferation was observed on PSS/PAH fibers compared to MH_6-coated substrates. Honey-coated fibers supported proliferation of primary endothelial cells with no significant differences depending on the concentration of honey. Proliferation was significantly lower on PSS/PAH and bare PCL samples compared to the maximum value obtained on MH_1.5- and MH_3-coated fibers (*p* < 0.05).

Finally, the antibacterial activity of MH against both Gram-positive and Gram-negative species was evaluated by the broth microdilution method. The assay showed an MIC at 200 mg/ml (13.6% *v*/*v*) for *S. aureus* and *S. epidermidis*, at 300 mg/ml (20.4% *v*/*v*) for *E. coli* and at 500 mg/ml (34% *v*/*v*) for *P. aeruginosa*. The antibacterial activity of 2 mg of each LbL-functionalized electrospun membrane was quantified by means of the MTT assay. Figure 9B shows the differences in the metabolic activity of cells coincubated for 24 h with the different types of membranes. In comparison with neat PCL membranes, PSS/PAH samples showed no antibacterial activity. On the other hand, the efficacy of honey-LbL-functionalized electrospun membranes depended on the content of MH and on the tested strain. Honey-functionalized membranes had no significant efficacy against the Gram-negative species *E. coli* and *P. aeruginosa* (averagely from 1.1 to 11.1% inhibition). Increasing but negligible inhibitions were observed against *S. aureus* for MH_1.5, MH_3, and MH_6 samples, whereas a significant reduction of 27.2% was detected for MH_12 (*p* < 0.001). For *S. epidermidis*, significant inhibitory activities of 13.5% for MH_3, of 23.4% for MH_6, and of 34.3% for MH_12 were found (*p* < 0.001).

## Discussion

Bacterial resistance to antimicrobial agents is an increasing health and economic problem (Frieri et al., 2017). As an alternative to antimicrobial drugs, the therapeutic use of ancient remedies has been revaluated in recent years. Honey is known for its therapeutic potential, including wound healing properties and antimicrobial activity. Since ancient times, honey has been a traditional remedy of several human diseases (Eteraf-Oskouei and Najafi, 2013). The antibacterial properties of honey are strongly influenced by its high osmolarity, acidity, content of hydrogen peroxide, and, in the specific case of MH, also by phytochemical components like methylglyoxal and leptosperin (Jenkins et al., 2015). However, toxic cellular effects of the honey-derived agents could potentially limit its clinical use. To date, only a few papers have shown the cytocompatibility of MH-based structures for tissue engineering applications, reporting the correlation of MH concentration with its toxicity to human cells. In this work, we proposed the following: (1) LbL assembly as an effective surface functionalization technology and (2) for the first time, MH as a PE within the LbL assembly strategy to confer antibacterial properties and preserve the cytocompatibility of PCL electrospun membranes. Among others, electrospinning technology offers the unique opportunity to develop ECM-like substrates, with biomimetic features, able to enhance soft tissue regeneration. In addition to this, as demonstrated recently by Gentile et al. (2015b), LbL technology allows the incorporation of a relatively small amount of loaded drug/biomolecules needed to achieve effective concentrations for a localized and controlled delivery system without affecting the physicochemical properties of the substrate.

Therefore, to consider MH as a novel PE, electrophoresis measurements were performed, and they showed that MH-based solutions were negatively charged and the MH trend was in accordance with previous studies, where the ζ-potential was significantly influenced by the concentration of different studied polysaccharides. Particularly, sodium alginate and k-carrageenan solutions, considered as weak PEs, showed that the increase in the polysaccharide concentrations lead to more negative ζ-potential values (Carneiro-da-Cunha et al., 2011). On the other hand, it was reported in the literature that strong PEs, such as polyethyleneimine, were characterized by an opposite trend, where an increase in the polymer concentration lead to lower ζ-potential values (Lindquist and Stratton, 1976). This indicates that MH acts as a partially weak PE as the natural-based PEs.

A confirmation of the MH potential within LbL functionalization was provided by QCM-D measurements, where MH and PAH demonstrated a stable electrostatic complexation with the tendency of PAH to form a more rigid layer in comparison to MH, as the dissipation factor underwent a more pronounced shift when MH layer was recorded (Deligöz and Tieke, 2014).

Biomimetic fiber-based membranes were produced through electrospinning to mimic the nanofibrous structure of the native ECM (Wang et al., 2013). Among other polymers, PCL was selected as former material, thanks to its well-known biocompatibility and its stability during LbL processing without compromising bulk material properties and structure morphology (Malikmammadov et al., 2018). For the optimal functionalization conditions of PCL-based electrospun membranes via LbL assembly, the process parameters were set as follows: a total number of 16 nanolayers, dipping time into the PE solutions of 15 min, PAH molar concentration of 0.5 M, in accordance to a previous work reported by the authors (Gentile et al., 2017). To favor the absorption of the first PE, aminolysis treatment was performed before the LbL assembly functionalization to incorporate primary and secondary NH_2_ groups onto the substrate (Ferreira et al., 2016). The surface functionalization did not influence the intrinsic microporosity of the electrospun membranes, which is fundamental for cell penetration, nutrient transport, and waste removal (Sell et al., 2008), and to make them available biocues of the native ECM (Wang et al., 2013). Particularly, SEM showed for the control PSS/PAH-coated membranes a formation of irregularly shaped protuberances and a consequently higher surface roughness and fiber diameter in comparison to the bare substrate. This is typical of strong complexation between these two PEs and becomes more evident with an increasing number of bilayers, as demonstrated by several studies (Park et al., 2008; Gribova et al., 2011). On the other hand, similarly to other natural PEs, MH coating appeared smooth and uniform, leading to an increase in the fiber diameter, which in turn resulted proportional to the number of bilayers used (Park et al., 2009; Gomes et al., 2015).

**Figure 10:**
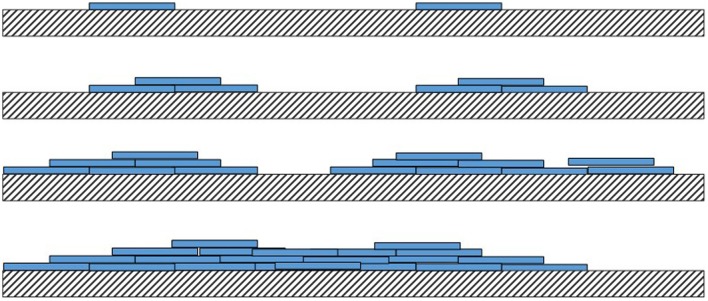
Schematic cartoon of the layer-by-layer (LbL) mechanism.

Although not quantitative, AFM tapping mode phase contrast signal can very clearly differentiate different material properties, and in our work, the coating substance showed a reduced phase lag (lighter in the images) compared with the PCL fiber (darker phase contrast). In phase contrast, PCL fibers showed typical striations (13.5 nm periodicity around the fiber circumference), which are the polymer crystalline lamellae of the PCL layered between amorphous regions, as observed previously in 100% PCL fibers with a spacing of 14.8 ± 2.9 nm (Goonoo et al., 2015), a value which increased upon addition of another polymer to the fiber composition. In most partially crystalline polymers, these lamellae typically lie in the range of 10–20 nm. They are more visible in the high-resolution AFM phase contrast images of the fibers (for PCL and MH_1.5). Furthermore, AFM indicated that the resultant concentration vs. coverage response was indicative of an absorption isotherm, where the progressive coverage with concentration indicates that absorption of the partially strong PE to this heterogeneous and porous surface follows a typical surface absorption mechanism, as well as being subject to LbL growth once the initial absorption site have taken hold. During the LbL process at a given concentration, these sites will gradually thicken and spread out filling in the remaining uncoated regions (for a cartoon schematic of this mechanism, see Figure 10).

In this work, we reported a lower MH-loaded content, comparing with other papers reported in the literature, with a more controlled release in the longer period. Hixon et al. reported a release of 0.5 mg/ml of MH for the first 24 h, followed by a slight decrease at 4 days and a sustained break down after 7 days (Hixon et al., 2017). In our work, we demonstrated that LbL assembly can be an effective methodology for having a controlled release of the natural antibacterial agent within 2 weeks, which represent the period in which most implant-associated infections are initiated (Birlutiu et al., 2017).

Finally, biological tests demonstrated that MH coating supported proliferation of different cell types (fibroblasts and endothelial cells) without compromising the biocompatibility of the meshes, while the antibacterial ones showed that the antimicrobial MH activity was dependent on the concentration used and the bacteria strain tested. In accordance with our observation, Bucekova et al. (2018) demonstrated that medical-grade MH was more effective against Gram-positive than Gram-negative bacteria. Similar results were shown by Tan et al. (2009) with MIC_95_ values of 12.5% (*v*/*v*) and 20% (*v*/*v*) for *S. aureus* and *E. coli*, respectively.

Furthermore, *E. coli* inhibition was demonstrated by means of the agar diffusion assay for PCL scaffolds containing 10 and 20% of MH by Guthrie et al. (2012). Yang et al. reported bacterial inhibition rates of different MH/silk fibroin fibrous matrices (Yang et al., 2017). In particular, for MH (10%)/silk fibroin, an inhibitory effect ranging from 5 to 10% was detected against *S. aureus, E. coli, P. aeruginosa*, and methicillin-resistant *S. aureus* strains. The observed dependence to the bacterial strain might be identified, at first, in the structural differences of their cell walls. In comparison to Gram-negative strains, in fact, Gram-positive strains do not have an outer membrane that offers protection to the peptidoglycan layer from lysozyme and other antimicrobial agents, making them easier to penetrate and damage (Johnston et al., 2018). Second, the different grade of susceptibility of bacterial strains might be also due to the different mechanisms of action involved in MH's antibacterial activity (Henriques et al., 2010). For example, for *S. aureus*, it was observed that MH interferes with the regular cell division process (Gomes et al., 2015). Conversely, for *P. aeruginosa*, it was observed that inhibitory concentrations cause the loss of cellular integrity, extensive cell lysis, and death (Henriques et al., 2011).

Overall, the advantages of the nanofunctionalization strategy described here are marked: (1) to allow the manufacturing of biomimetic systems with expectable functional properties without compromising the physicochemical properties of the electrospun membranes; (2) *in vitro* release demonstrated that the MH-loaded meshes were capable of effectively delivering MH in a controlled way within 2–3 weeks of incubation; and (3) *in vitro* tests confirmed the cytocompatibility of all the proposed MH-based systems while the antibacterial ones showed that the antimicrobial MH activity was dependent on the concentration used and the bacteria strain tested. This study has therefore demonstrated that the combination of naturally derived antibacterial agent with LbL technique may be applied to the manufacture of medical devices with advanced functionality.

## Data Availability Statement

The raw data supporting the conclusions of this manuscript will be made available by the authors, without undue reservation, to any qualified researcher.

## Author Contributions

EM and PG conceived the study. EM, LF, SC, and PG designed the experiments. EM and PG performed the LbL functionalization. CT-T produced the membranes and performed the QCM. SC performed the AFM. EM performed the SEM. VP performed the *in vitro* cell tests. CC and LF performed the bacterial tests. All authors analyzed and interpreted the data and prepared the manuscript.

### Conflict of Interest

The authors declare that the research was conducted in the absence of any commercial or financial relationships that could be construed as a potential conflict of interest.

## References

[B1] Anjos O. Campos M. G. Ruiz P. C. Antunes P. (2015). Application of FTIR-ATR spectroscopy to the quantification of sugar in honey. Food Chem. 169, 218–223. 10.1016/j.foodchem.2014.07.138 25236219

[B2] Armstrong D. G. (2009). Manuka honey improved wound healing in patients with sloughy venous leg ulcers. Evid. Based Med. 14:148. 10.1136/ebm.14.5.148 19794023

[B3] Birlutiu R. M. Birlutiu V. Mihalache M. Mihalache C. Cismasiu R. S. (2017). Diagnosis and management of orthopedic implant-associated infection: a comprehensive review of the literature. Biomed. Res. 28, 5063–5073.

[B4] Bonifacio M. A. Cometa S. Cochis A. Gentile P. Ferreira A. M. Azzimonti B.. (2018). Antibacterial effectiveness meets improved mechanical properties: Manuka honey/gellan gum composite hydrogels for cartilage repair. Carbohyd. Polymers 198, 462–472. 10.1016/j.carbpol.2018.06.115 30093023

[B5] Bucekova M. Buriova M. Pekarik L. Majtan V. Majtan J. (2018). Phytochemicals-mediated production of hydrogen peroxide is crucial for high antibacterial activity of honeydew honey. Sci. Rep. 8:9061. 10.1038/s41598-018-27449-3 29899462 PMC5998132

[B6] Carneiro-da-Cunha M. G. Cerqueira M. A. Souza B. W. S. Teixeira J. A. Vicente A. A. (2011). Influence of concentration, ionic strength and pH on zeta potential and mean hydrodynamic diameter of edible polysaccharide solutions envisaged for multinanolayered films production. Carbohyd. Polymers 85, 522–528. 10.1016/j.carbpol.2011.03.001

[B7] Cokcetin N. N. Pappalardo M. Campbell L. T. Brooks P. Carter D. A. Blair S. E.. (2016). The antibacterial activity of Australian Leptospermum honey correlates with methylglyoxal levels. PLoS ONE 11:e0167780. 10.1371/journal.pone.0167780 28030589 PMC5193333

[B8] Dai Z. Ronholm J. Tian Y. Sethi B. Cao X. (2016). Sterilization techniques for biodegradable scaffolds in tissue engineering applications. J. Tissue Eng. 7:2041731416648810. 10.1177/2041731416648810 27247758 PMC4874054

[B9] Deligöz H. Tieke B. (2014). QCM-D study of layer-by-layer assembly of polyelectrolyte blend films and their drug loading-release behavior. Col. Surf. A Physicochem. Eng. Aspects 441, 725–736. 10.1016/j.colsurfa.2013.10.033

[B10] Du Toit D. F. Page B. J. (2009). An *in vitro* evaluation of the cell toxicity of honey and silver dressings. J. Wound Care 18, 383–389. 10.12968/jowc.2009.18.9.44307 19789475

[B11] Esposito S. Bassetti M. Concia E. De Simone G. De Rosa F. G. Grossi P.. (2017). Diagnosis and management of skin and soft-tissue infections (SSTI). A literature review and consensus statement: an update. J. Chemother. 29, 197–214. 10.1080/1120009X.2017.1311398 28378613

[B12] Eteraf-Oskouei T. Najafi M. (2013). Traditional and modern uses of natural honey in human diseases: a review. Iran. J. Basic Med. Sci. 16, 731–742. 23997898 PMC3758027

[B13] Ferreira A. M. Gentile P. Toumpaniari S. Ciardelli G. Birch M. A. (2016). Impact of collagen/heparin multilayers for regulating bone cellular functions. ACS Appl. Mater. Interfaces 8, 29923–29932. 10.1021/acsami.6b09241 27762547

[B14] Ferreira A. M. Tonda-Turo C. Mancuso E. Gentile P. (2019). Multilayer nanoscale functionalisation to treat disorders and enhance regeneration of bone tissue. Nanomedicine 19:22–38. 10.1016/j.nano.2019.03.009 31002932

[B15] Frieri M. Kumar K. Boutin A. (2017). Antibiotic resistance. J. Infect. Public Health 10, 369–378. 10.1016/j.jiph.2016.08.007 27616769

[B16] Gentile P. Carmagnola I. Nardo T. Chiono V. (2015a). Layer-by-layer assembly for biomedical applications in the last decade. Nanotechnology 26:422001. 10.1088/0957-4484/26/42/422001 26421916

[B17] Gentile P. Ferreira A. M. Callaghan J. T. Miller C. A. Atkinson J. Freeman C.. (2017). Multilayer nanoscale encapsulation of biofunctional peptides to enhance bone tissue regeneration *in vivo*. Adv. Healthcare Mater. 6:1601182. 10.1002/adhm.201601182 28169513

[B18] Gentile P. Frongia M. E. Cardellach M. Miller C. A. Stafford G. P. Leggett G. J.. (2015b). Functionalised nanoscale coatings using layer-by-layer assembly for imparting antibacterial properties to polylactide-co-glycolide surfaces. Acta Biomater. 21, 35–43. 10.1016/j.actbio.2015.04.009 25871538

[B19] Gomes A. P. Mano J. F. Queiroz J. A. Gouveia I. C. (2015). Layer-by-layer assembly for biofunctionalization of cellulosic fibers with emergent antimicrobial agents, in Cellulose Chemistry and Properties: Fibers, Nanocelluloses and Advanced Materials ed Rojas O. J. (Raleigh, NC: Springer), 225–240. 10.1007/12_2015_318

[B20] Goonoo N. Bhaw-Luximon A. Rodriguez I. A. Wesner D. Schönherr H. Bowlin G. L. (2015). Poly (ester-ether) s: III. assessment of cell behaviour on nanofibrous scaffolds of PCL, PLLA and PDX blended with amorphous PMeDX. J. Mater. Chem. B, 3, 673–687. 10.1039/C4TB01350F 32262350

[B21] Gribova V. Auzely-Velty R. Picart C. (2011). Polyelectrolyte multilayer assemblies on materials surfaces: from cell adhesion to tissue engineering. Chem. Mater. 24, 854–869. 10.1021/cm2032459 25076811 PMC4112380

[B22] Guthrie K. M. Agarwal A. Tackes D. S. Johnson K. W. Abbott N. L. Murphy C. J.. (2012). Antibacterial efficacy of silver-impregnated polyelectrolyte multilayers immobilized on a biological dressing in a murine wound infection model. Ann. Surg. 256, 371–377. 10.1097/SLA.0b013e318256ff99 22609841 PMC3433034

[B23] Henriques A. F. Jenkins R. E. Burton N. F. Cooper R. A. (2010). The intracellular effects of manuka honey on *Staphylococcus aureus*. Eur. J. Clin. Microbiol. Infect. Dis. 29, 45–50. 10.1007/s10096-009-0817-2 19813035

[B24] Henriques A. F. Jenkins R. E. Burton N. F. Cooper R. A. (2011). The effect of manuka honey on the structure of *Pseudomonas aeruginosa*. Eur. J. Clin. Microbiol. Infect. Dis. 30, 167–171. 10.1007/s10096-010-1065-1 20936493

[B25] Hixon K. R. Lu T. McBride-Gagyi S. H. Janowiak B. E. Sell S. A. (2017). A comparison of tissue engineering scaffolds incorporated with Manuka honey of varying UMF. BioMed Res. Int. 2017:4843065. 10.1155/2017/4843065 28326322 PMC5343224

[B26] Jenkins R. Roberts A. Brown H. L. (2015). On the antibacterial effects of manuka honey: mechanistic insights. Res. Rep. Biol. 6, 215–224. 10.2147/RRB.S75754

[B27] Johnston M. McBride M. Dahiya D. Owusu-Apenten R. Nigam P. S. (2018). Antibacterial activity of Manuka honey and its components: an overview. AIMS Microbiol. 4:655. 10.3934/microbiol.2018.4.655 31294240 PMC6613335

[B28] Kruk T. Szczepanowicz K. Kregiel D. Szyk-Warszynska L. P. (2016). Warszynski: Nanostructured multilayer polyelectrolyte films with silver nanoparticles as antibacterial coatings. Coll. Surf. B Biointerfaces 137, 158–166. 10.1016/j.colsurfb.2015.06.016 26193773

[B29] Kumar V. R. M. N. (2000). A review of chitin and chitosan applications. Reactive Funct. Polymers 46, 1–27. 10.1016/S1381-5148(00)00038-9

[B30] Li B. Webster T. J. (2018). Bacteria antibiotic resistance: new challenges and opportunities for implant-associated orthopedic infections. J. Orthopaed. Res. 36, 22–32. 10.1002/jor.23656 28722231 PMC5775060

[B31] Lindquist G. M. Stratton R. A. (1976). The role of polyelectrolyte charge density and molecular weight on the adsorption and flocculation of colloidal silica with polyethylenimine. J. Colloid Interface Sci. 55, 45–59. 10.1016/0021-9797(76)90007-2

[B32] Malikmammadov E. Tanir T. E. Kiziltay A. Hasirci V. Hasirci N. (2018). PCL and PCL-based materials in biomedical applications. J. Biomater. Sci. Polymer Ed. 29, 863–893. 10.1080/09205063.2017.1394711 29053081

[B33] Maxson T. Mitchell D. A. (2016). Targeted treatment for bacterial infections: prospects for pathogen-specific antibiotics coupled with rapid diagnostics. Tetrahedron 72:3609. 10.1016/j.tet.2015.09.069 27429480 PMC4941824

[B34] Miller L. G. Eisenberg D. F. Liu H. Chang C.-L. Wang Y. Luthra R.. (2015). A. Incidence of skin and soft tissue infections in ambulatory and inpatient settings, 2005–2010. BMC Infect. Dis. 15:362. 10.1186/s12879-015-1071-0 26293161 PMC4546168

[B35] Minden-Birkenmaier B. Bowlin G. (2018). Honey-based templates in wound healing and tissue engineering. Bioengineering 5:46. 10.3390/bioengineering5020046 29903998 PMC6027142

[B36] Molan P. C. (2001). Honey as a topical antibacterial agent for treatment of infected wounds. World Wide Wounds 10.

[B37] Park J. H. Kim B. S. Tae H. J. Kim I. S. Kim H. Y. Khi M. S. (2009). Polyelectrolyte multilayer coated nanofibrous mats: Controlled surface morphology and cell culture. Fibers Polymers 10, 419–424. 10.1007/s12221-009-0419-8

[B38] Park J. H. Kim B. S. Yoo Y. C. Khil M. S. Kim H. Y. (2008). Enhanced mechanical properties of multilayer nano-coated electrospun nylon 6 fibers via a layer-by-layer self-assembly. J. Appl. Polymer Sci. 107, 2211–2216. 10.1002/app.27322

[B39] Park S. Han U. Choi D. Hong J. (2018). Layer-by-layer assembled polymeric thin films as prospective drug delivery carriers: design and applications. Biomater. Res. 22:29. 10.1186/s40824-018-0139-5 30275972 PMC6158909

[B40] Pfalzgraff A. Brandenburg K. Weindl G. (2018). Antimicrobial peptides and their therapeutic potential for bacterial skin infections and wounds. Front. Pharmacol. 9:281. 10.3389/fphar.2018.00281 29643807 PMC5882822

[B41] Qi L. Knapton E. K. Zhang X. Zhang T. Gu C. Zhao Y. (2017). Pre-culture Sudan Black B treatment suppresses autofluorescence signals emitted from polymer tissue scaffolds. Sci. Rep. 7:8361. 10.1038/s41598-017-08723-2 28827657 PMC5567053

[B42] Ratkowsky D. A. Giles D. E. (1990). A Handbook of Nonlinear Regression Models. Ann Arbor, MI: University of Michigan; Marcel Dekker Inc.

[B43] Richardson J. J. Björnmalm M. Caruso F. (2015). Technology-driven layer-by-layer assembly of nanofilms. Science 348:aaa2491. 10.1126/science.aaa2491 25908826

[B44] Rojas O. J. Claesson P. M. Muller D. Neuman R. D. (1998). The effect of salt concentration on adsorption of low-charge-density polyelectrolytes and interactions between polyelectrolyte-coated surfaces. J. Coll. Interface Sci. 205, 77–88. 10.1006/jcis.1998.5596 9710501

[B45] Roy R. Tiwari M. Donelli G. Tiwari V. (2018). Strategies for combating bacterial biofilms: a focus on anti-biofilm agents and their mechanisms of action. Virulence 9, 522–554. 10.1080/21505594.2017.1313372 28362216 PMC5955472

[B46] Sadeghi-Aliabadi H. Hamzeh J. Mirian M. (2015). Investigation of Astragalus honey and propolis extract's cytotoxic effect on two human cancer cell lines and their oncogen and proapoptotic gene expression profiles. Adv. Biomed. Res. 4:42. 10.4103/2277-9175.151251 25789268 PMC4358038

[B47] Sell S. Barnes C. Simpson D. Bowlin G. (2008). Scaffold permeability as a means to determine fiber diameter and pore size of electrospun fibrinogen. J. Biomed. Mater. Res. A 85, 115–126. 10.1002/jbm.a.31556 17688269

[B48] Séon L. Lavalle P. Schaaf P. Boulmedais F. (2015). Polyelectrolyte multilayers: a versatile tool for preparing antimicrobial coatings. Langmuir 31, 12856–12872. 10.1021/acs.langmuir.5b02768 26513437

[B49] Tan H. T. Rahman R. A. Gan S. H. Halim A. S. Asma'Hassan S. Sulaiman S. A. B. S.. (2009). Kirnpal-Kaur: the antibacterial properties of Malaysian tualang honey against wound and enteric microorganisms in comparison to manuka honey. BMC Complement. Altern. Med. 9:34. 10.1186/1472-6882-9-34 19754926 PMC2753561

[B50] Tonda-Turo C. Carmagnola I. Ciardelli G. (2018b). Quartz crystal microbalance with dissipation monitoring: a powerful method to predict the *in vivo* behavior of bioengineered surfaces. Front. Bioeng. Biotechnol. 6:158. 10.3389/fbioe.2018.00158 30425985 PMC6218436

[B51] Tonda-Turo C. Ruini F. Ceresa C. Gentile P. Varela P. Ferreira A. M.. (2018a). Nanostructured scaffold with biomimetic and antibacterial properties for wound healing produced by ‘green electrospinning’. Coll. Surf. B Biointerface, 172, 233–243. 10.1016/j.colsurfb.2018.08.039 30172204

[B52] Tun K. Shurko J. F. Ryan L. Lee G. C. (2018). Age-based health and economic burden of skin and soft tissue infections in the United States, 2000 and 2012. PLoS ONE 13:e0206893. 10.1371/journal.pone.0206893 30383858 PMC6211756

[B53] Wang X. Ding B. Li B. (2013). Biomimetic electrospun nanofibrous structures for tissue engineering. Mater. Tdy 16, 229–241. 10.1016/j.mattod.2013.06.005 25125992 PMC4130655

[B54] Wiegand I. Hilpert K. Hancock R. E. (2008). W. Agar and broth dilution methods to determine the minimal inhibitory concentration (MIC) of antimicrobial substances. Nat. Protoc. 3:163 10.1038/nprot.2007.521 18274517

[B55] Yang X. Fan L. Ma L. Wang Y. Lin S. Yu F. (2017). Green electrospun Manuka honey/silk fibroin fibrous matrices as potential wound dressing. Mater. Design, 119, 76–84. 10.1016/j.matdes.2017.01.023

[B56] Zahin N. Anwar R. Tewari D. Kabir M. T. Sajid A. Mathew B.. (2019). Nanoparticles and its biomedical applications in health and diseases: special focus on drug delivery. Environ. Sci. Pollut. Res. 26, 1–18. 10.1007/s11356-019-05211-0 31079299

[B57] Zhang L. Huang Y. Zhou Y. Buckley T. Wang H. H. (2013). Antibiotic administration routes significantly influence the levels of antibiotic resistance in gut microbiota. Antimicrob. Agents Chemother. 57, 3659–3666. 10.1128/AAC.00670-13 23689712 PMC3719697

[B58] Zhu T. Sha Y. Yan J. Pageni P. Rahman M. A. Yan Y.. (2018). Metallo-polyelectrolytes as a class of ionic macromolecules for functional materials. Nat. Commun. 9:4329. 10.1038/s41467-018-06475-9 30337530 PMC6193978

[B59] Zimlichman E. Henderson D. Tamir O. Franz C. Song P. Yamin C. K.. (2013). Health care–associated infections: a meta-analysis of costs and financial impact on the US health care system. JAMA Internal Med. 173, 2039–2046. 10.1001/jamainternmed.2013.9763 23999949

[B60] Zinoviadou K. G. Koutsoumanis K. P. Biliaderis C. G. (2009). Physico-chemical properties of whey protein isolate films containing oregano oil and their antimicrobial action against spoilage flora of fresh beef. Meat Sci. 82, 338–345. 10.1016/j.meatsci.2009.02.004 20416718

